# Dietary Folate Intake and Folic Acid Supplements among Pregnant Women from Southern Italy: Evidence from the “Mamma & Bambino” Cohort

**DOI:** 10.3390/ijerph17020638

**Published:** 2020-01-19

**Authors:** Martina Barchitta, Andrea Maugeri, Roberta Magnano San Lio, Giuliana Favara, Claudia La Mastra, Maria Clara La Rosa, Antonella Agodi

**Affiliations:** Department of Medical and Surgical Sciences and Advanced Technologies “GF Ingrassia”, University of Catania, Via S. Sofia 87, 95123 Catania, Italy; martina.barchitta@unict.it (M.B.); andrea.maugeri@unict.it (A.M.); robimagnano@gmail.com (R.M.S.L.); giuliana.favara@gmail.com (G.F.); claudia.lamastra@libero.it (C.L.M.); mariclalarosa@gmail.com (M.C.L.R.)

**Keywords:** pregnancy, nutrition, folate, neonatal outcomes, public health

## Abstract

Folate requirement among women who plan to become pregnant should be raised to 600 μg/day during the periconceptional period. To meet this need, several countries began to promote the use of folic acid supplements before and during pregnancy. Here, we investigated prevalence and determinants of dietary folate intake and folic acid supplement use among 397 pregnant women (aged 15–50 years old, median = 37 years old). We also investigated their effects on neonatal outcomes in a subgroup of women who completed pregnancy. For doing that, we used data from the “Mamma & Bambino” project, an ongoing mother-child cohort settled in Catania (Italy). Inadequate folate intake was evaluated using a Food Frequency Questionnaire and defined as an intake < 600 μg/day. Women were also classified as non-users (i.e., women who did not use folic acid supplements), insufficient users (i.e., women who did not take folic acid supplements as recommended), and recommended users of folic acid supplements. Neonatal outcomes of interest were preterm birth (PTB) and small for gestational age (SGA). Nearly 65% of women (*n* = 257) reported inadequate folate intake, while 74.8% and 22.4% were respectively classified as insufficient or recommended users of supplements. We demonstrated higher odds of inadequate folate intake among smoking women (OR = 1.457; 95%CI = 1.046–2.030; *p* = 0.026), those who followed dietary restrictions (OR = 2.180; 95%CI = 1.085–4.378; *p* = 0.029), and those with low adherence to the Mediterranean Diet (OR = 3.194; 95%CI = 1.958–5.210; *p* < 0.001). In a subsample of 282 women who completed pregnancy, we also noted a higher percentage of SGA among those with inadequate folate intake (*p* < 0.001). Among 257 women with inadequate folate intake, those with low educational level were more likely to not take folic acid supplements than their more educated counterpart (OR = 5.574; 95%CI = 1.487–21.435; *p* = 0.012). In a subsample of 184 women with inadequate folate intake and complete pregnancy, we observed a higher proportion of SGA newborns among women who did not take supplement before pregnancy and those who did not take at all (*p* = 0.009). We also noted that the proportion of PTB was higher among non-users and insufficient users of folic acid supplements, but difference was not statistically significant. Our study underlined the need for improving the adherence of pregnant women with recommendations for dietary folate intake and supplement use. Although we proposed a protective effect of folic acid supplement use on risk of SGA, further research is encouraged to corroborate our findings and to investigate other factors involved.

## 1. Introduction

During pregnancy, maternal nutrition plays a key role in fetal development and neonatal growth [1]. Specifically, during the preconception and gestational periods, inadequate intake of micronutrients might affect the risk of adverse pregnancy outcomes [2]. In line, mounting evidence suggests a strictly interplay between newborn and mother metabolisms, which in turn involve nutrient stores and intakes [3,4,5]. For this reason, the World Health Organization (WHO) and the Food and Agricultural Organization (FAO) developed several dietary recommendations and strategies for the prevention of adverse pregnancy outcomes [6]. Among these, pre-term birth (PTB; birth before 37 weeks of gestation) and small for gestational age (SGA; birth weight below the 10th percentile for gestational age) represent the major causes of death among newborns [7,8,9].

Folate—a water-soluble vitamin B found in fruits, legumes, cereals, and green leafy vegetables—is required for placental tissue growth [10] and neural tube formation [11]. More recently, its role as a methyl donor in several molecular pathways and epigenetic mechanisms has been demonstrated [12,13]. Folate requirement among women of childbearing age is usually of 400 μg/day, but it should be raised to 600 μg/day during the periconceptional period [14]. To meet this need, in 1998, the United States began the compulsory fortification of cereal flour enriched with folic acid [15]. Subsequently, several countries promoted policies for folate fortification [16]. Nowadays, developed countries proposed folic acid supplementation as a strategy to ensure the correct fetal growth [17]. However, several lines of evidence showed that folic acid supplementation was often insufficient in the preconception period, with several negative effects on pregnancy and neonatal outcomes [18].

Thus, further studies should investigate social and behavioral determinants that might affect the adherence to these recommendations and increase the awareness about benefits of folic acid supplement use [19,20,21,22]. Although inadequate folate concentrations were often associated with anencephaly and spina bifida [23], its impact on other adverse pregnancy outcomes is not fully understood. Interestingly, there was also evidence that folate status and supplement use were associated with a slightly increased risk for wheeze and lower respiratory tract infections in newborns [24,25,26].

Our hypothesis is that folate deficiency leads to PTB [27] and SGA [28,29,30,31,32,33,34,35,36,37,38,39,40,41,42,43,44,45,46], but further studies are necessary to better investigate the potential protective role of adequate folate intake and/or folic acid supplementation. To fill this gap, the primary aim of the current study was to describe the prevalence of dietary folate intake and its determinants among pregnant women from Catania (Italy). We also evaluated folic acid supplement use according to national recommendations. Finally, we investigated the effect of folate intake and folic acid supplement use on neonatal outcomes in a subgroup of women who completed pregnancy.

## 2. Materials and Methods

### 2.1. Study Design

In the current analysis, we used data from the Mamma & Bambino project, an ongoing mother-child cohort settled in Catania, Italy, which aims to understand the effects of social, environmental, behavioral, and molecular factors on maternal and infant health. Study design and protocols have been fully described elsewhere [47,48] and at the website http://www.birthcohorts.net. From 2015, this cohort prospectively recruits pregnant women during their prenatal genetic counselling (median gestational age = 16 weeks; range = 4–20 weeks) at the Azienda Ospedaliera Universitaria “Policlinico-Vittorio Emanuele” (Catania, Italy). Pregnancy is the unit of observation with planned follow-up of children at birth, one and two years. Women with plurality, pre-existing medical conditions or pregnancy complications (i.e., autoimmune and/or chronic diseases, preeclampsia, gestational hypertension, and diabetes), intrauterine fetal death, and congenital malformations were excluded from this study. The study protocol has been approved by the ethics committee of the involved Institution (CE Catania 2; Prot. N. 227/BE and 275/BE). Participants were informed and gave written their informed consent of the purpose and procedures of the study, which was conducted according to the Declaration of Helsinki.

### 2.2. Data Collection

At the recruitment, socio-demographic and behavioral information was collected by trained epidemiologist through structured questionnaires. Educational level was classified as low (primary school), medium (high school), or high (degree or higher), while employment status was categorized as unemployment (including students and housewives) and employment (both part-time and full-time). Women were also classified in those who lived alone or in couple, and in those who had children or not. With respect to smoking status, women were classified as non-smokers, former smokers, or current smokers. Women were asked to report their weight and height before pregnancy and pregestational BMI was calculated as weight in kg divided by height in m^2^ and classified according to the WHO criteria [49]. Women were also asked to report if they followed dietary restrictions or suffered from food intolerances.

### 2.3. Assessment of Dietary Folate Intake and Adherence to Mediterranean Diet

Dietary folate intake was evaluated using a 95-item semi-quantitative Food Frequency Questionnaire (FFQ) referred to 30 days before recruitment [12,13,50,51,52] and hence to the early phase of pregnancy (i.e., from the beginning to the 16 week of gestation). The extended form of this tool was adapted from a 46-item FFQ, which has been validated for the assessment of folate intake in Italian women of child-bearing age [13]. For each food item, information on frequency of consumption (twelve categories from “almost never” to “two or more times a day”) and portion size (small, medium, and large) were collected using an indicative photograph atlas, and then converted into daily food intakes. Dietary folate intake was calculated using the table of food composition of the US Department of Agriculture (http://ndb.nal.usda.gov/), adapted to typical Italian food consumption. Inadequate folate intake was defined as an intake < 600 μg/day of dietary folate equivalents (DFEs) [53]. Adherence to the Mediterranean Diet (MD) was evaluated using the 9-item Mediterranean Diet Score (MDS), as previously described [54,55]. For this reason, ranged from 0 (non-adherence) to 9 (perfect adherence) and adherence to MD was classified as low (MDS ≤ 3), medium (MDS = 4–6), or high (MDS > 6) [56].

### 2.4. Use of Folic Acid Supplements

Women were also asked to report the use of folic acid supplements, alone or in combination with other multivitamin supplements, before pregnancy and during the first trimester of pregnancy. The current Italian recommendation suggests that women who plan to become pregnant should use folic acid supplements for 4 weeks before and until 12 weeks after conception [57]. Accordingly, women were classified as non-users (i.e., women who did not use folic acid supplements), insufficient users (i.e., women who did not take folic acid supplements as recommended), and recommended users.

### 2.5. Neonatal Outcomes

Gestational age and neonatal anthropometric measures were assessed at birth, among women with who completed singleton pregnancy. At recruitment, gestational age was assessed by ultrasound evaluation and used to define preterm birth as spontaneous delivery before 37 weeks. According to sex-specific national reference charts, birth weight and length were used to assess birthweight for gestational as follows: small for gestational age (birth weight < 10th percentile for gestational age), adequate for gestational age, or large for gestational age (birth weight > 90th percentile for gestational age) [58].

### 2.6. Statistical Analysis

Statistical analyses were performed using SPSS software version 26.0 (SPSS, Chicago, IL, USA). Characteristics of pregnant women according to dietary folate intake and folic acid supplement use were described using frequency (%) or median and interquartile range (IQR). Categorical variables were compared using Chi-squared test. Continuous variables were checked for normality using the Kolmogorov-Smirnov test and compared using the Mann-Whitney U test. Logistic regression analysis was used to identify main determinants of inadequate folate intake and folic acid supplement use. The models included variables that were significantly associated with inadequate folate intake or folic acid supplement use in the univariate analysis. Results were reported as Odds ratio (OR) and 95% confidence interval (CI). All statistical tests were two-sided, and *p*-values < 0.05 were considered statistically significant.

## 3. Results

### 3.1. Dietary Folate Intake Among Pregnant Women

**Figure 1:**
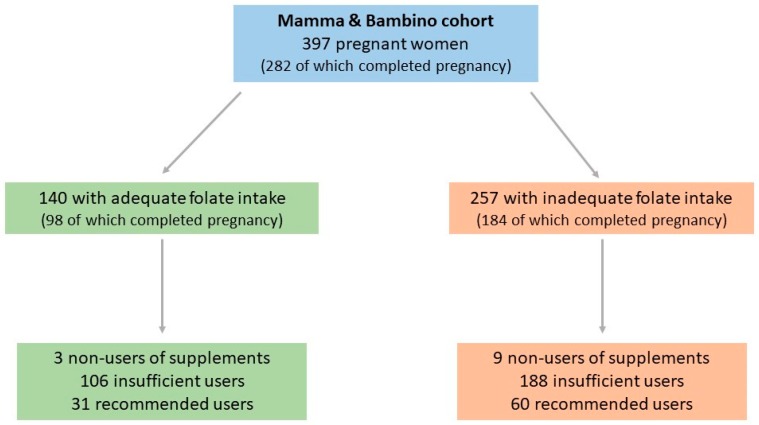
The distribution of women according to dietary folate intake and use of supplements.

The current study included 397 pregnant women from the “Mamma & Bambino” cohort (aged 15–50 years old, median = 37 years old) recruited from 2015 to 2019, 282 out of which completed pregnancy at the time of this study. In general, the average dietary folate intake was 533.4 μg/day (median = 516.3 μg/day; range = 68.9–2633.5 μg/day), and 64.7% of women (n = 257) did not meet the current recommendation of 600 μg/day during pregnancy. Figure 1 displays the distribution of women according to dietary folate intake and the use of supplements. Table 1 shows the characteristics of women according to their dietary folate intake. We observed that women who did not meet dietary recommendation were more likely to be smokers (*p* = 0.028) and exhibited higher pregestational BMI (*p* = 0.029) than their counterpart. With respect to dietary habits, women who did not meet dietary recommendation were more likely to follow dietary restrictions (*p* = 0.003) and less likely to adhere to MD (*p* < 0.001). Interestingly, logistic regression analysis demonstrated that following dietary restrictions (OR = 2.180; 95%CI = 1.085–4.378; *p* = 0.029), being a smoker (OR = 1.457; 95%CI = 1.046–2.030; *p* = 0.026), and low adherence to MD (OR = 3.194; 95%CI = 1.958–5.210; *p* < 0.001) were the main determinants of inadequate folate intake. In the subsample of 282 women who completed pregnancy, we also noted a higher percentage of SGA and LGA among those with inadequate folate intake (*p* < 0.001).

### 3.2. Use of Folic Acid Supplements

**Figure 2:**
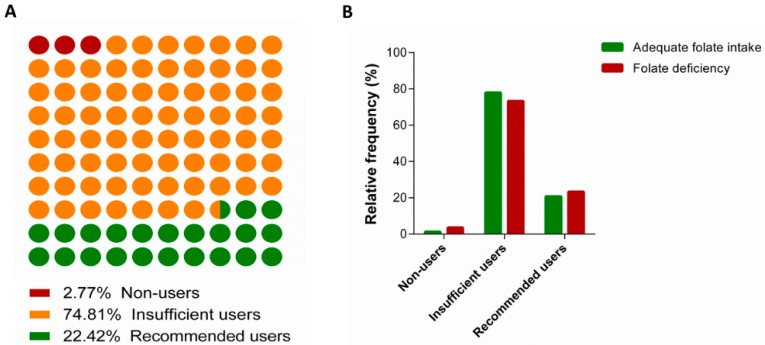
Use of folic acid supplements among pregnant women. (**A**) Panel A shows proportions of non-users, insufficient users and recommended users among the overall cohort. (**B**) Panel B shows the categories of folic acid supplement according to folate intake.

We next examined the use of folic acid supplements among women of the Mamma & Bambino cohort. Figure 2A shows that only 2.8% of women did not take supplements, 74.8% were classified as insufficient users before pregnancy, while 22.4% met the recommendation before and during pregnancy. We also compared supplement use between women with inadequate folate intake and those with adequate folate intake (Figure 2B). Compared with the latter, we observed higher proportions of non-users and recommended users among women with inadequate folate intake. By contrast, a higher proportion of insufficient users has been observed among women with adequate dietary folate intake. However, these differences were not statistically significant. With respect to neonatal outcomes, we did not observe differences in the proportion of preterm birth and inadequate birthweight for gestational age (*p* = 0.430 and *p* = 0.770, respectively).

### 3.3. Determinants of Folic Acid Supplement USE Among Women with Inadequate Folate Intake

We next aimed to identify the main determinants of folic acid supplement use among 257 women who did not meet the recommendation of dietary folate intake. Univariate analysis showed that women who did not take supplements were less educated (*p* < 0001) and reported lower MDS (*p* = 0.047) than supplement users (Table 2). Notably, logistic regression analysis further confirmed that women with low educational level were more likely to not take folic acid supplements than their more educated counterpart (OR = 5.574; 95%CI = 1.487–21.435; *p* = 0.012).

### 3.4. Use of Supplements and Neonatal Outcomes

**Figure 3:**
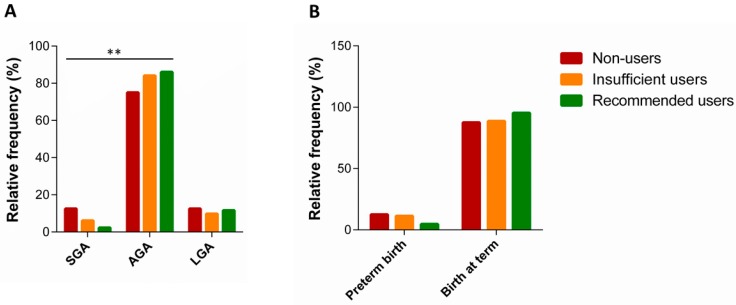
Neonatal adverse outcomes and folic acid supplement use among women with folate deficiency. (**A**) Panel A shows the distribution of small for gestational age (SGA), adequate for gestational age (AGA) and large for gestational age (LGA) infants. (**B**) Panel B shows the distribution of preterm and at term birth. ** *p*-value < 0.01.

Finally, we examined the effect of folic acid supplement use on neonatal outcomes among women with inadequate folate intake. With this in mind, we investigated 184 women with inadequate folate intake, who completed singleton pregnancy. In this subsample, median gestational duration was 39 weeks, with 9.8% of preterm deliveries. With respect to neonatal anthropometric measures, median values of birth weight and length were 3.25 Kg (range = 1.0–4.75 Kg) and 50.0 cm (range = 41–56 cm), respectively. According to sex-specific national reference charts [51], approximately 84.1% of newborns were adequate for gestational age (AGA), while 5.5% and 10.4% have been classified as SGA or LGA, respectively. Compared with women who met supplement recommendation, we observed a higher proportion of SGA newborns among those who did not take supplements before pregnancy and those who did not take any at all (*p* = 0.009) (Figure 3A). By contrast, the proportion of AGA newborns was the highest among women who took supplements before and during pregnancy. Instead, no difference in the distribution of LGA newborns was evident. We also noted that the proportion of preterm newborns was higher among non-users and insufficient users of folic acid supplements (Figure 3B). However, these differences were not statistically significant.

## 4. Discussion

The recommended intake of folate for pregnant women is 600 μg/day DFE, an estimation that was introduced to account for difference in the bioavailability between synthetic folic acid and naturally occurring folate [53]. Magnitude of folate deficiency varies between and within countries, with higher prevalence in those without folic acid fortification of cereal-grain products [59,60]. In our study, two out of three women did not meet current recommendation. The high prevalence of deficient women was in line with figures obtained by previous studies from the same Italian region [12,13,50,61,62].

The primary aim of our study was to uncover the main determinants of dietary folate intake, an approach that could help the development of public health strategies against folate deficiency in states of increased demand (e.g., pregnancy and lactation). With this in mind, we observed that women who followed dietary restrictions and those with low adherence to MD were more likely to report inadequate folate intake. In fact, naturally-occurring folates are present in high concentrations in green leafy vegetables, dark green vegetables, legumes, and some fruits [63], so that higher intakes can be expected among people who follow a varied and balanced diet such as the MD. Moreover, we noted that inadequate folate intake was higher among current smokers than in former or non-smoking women. This is in line with previous findings reporting unhealthy diet among smokers [64,65,66], with greater intake of saturated fat and cholesterol, and lower intake of vitamins and fiber [67].

Beyond folate intake, supplementation of folic acid during the periconceptional period represents one of the best strategies to tackle pregnancy adverse outcomes, as suggested by the WHO in 2006 [17]. However, the prevalence of folic acid supplementation remains often inadequate in several countries [20,68]. Although we showed that only ~3% of pregnant women did not take folic acid supplements, ~75% of them did not take supplements as recommended (i.e., 4 weeks before conception until 8 weeks after). Our data were consistent with a previous study reporting that only 3% of Italian pregnant women used folic acid supplements as recommended [69]. By contrast, in other European countries, prevalence of recommended users reached 50% [70]. In our study, the proportions of insufficient or non-users were not significantly different according to dietary folate status. However, among women with inadequate folate intake, those with low educational level were more likely to not use folic acid supplements than their more educated counterpart. Several studies aimed to identify the main determinants of inadequate supplement use during pregnancy. Among social factors, for instance, it has been demonstrated that younger age [20], low income [71], educational level [68], and employment status [21] might affect the use of folic acid supplements. In our opinion, social inequalities in the use of supplements could be partially explained by the reduced level of knowledge, attitude, and awareness among the more disadvantaged groups [22]. Our findings, together with those from previous studies, underline the need for increasing the prevalence of folic acid supplementation through the identification of people at the highest risk for folate deficiency.

It has been clearly demonstrated that low maternal folate intake during the periconceptional period increases the risk for neural tube defect (e.g., spina bifida, anencephaly) and perhaps for other congenital anomalies (e.g., congenital heart defects, oral cleft lip and plate) and adverse outcomes [17]. In our study, for instance, we reported higher proportions of SGA and LGA births among women with inadequate folate intake compared with those who met dietary recommendation. However, these data did not take into account the use of folic acid supplements. For this reason, we also evaluated the effects of folic acid supplements among women with inadequate folate intake. In this subgroup, we consistently reported a higher proportion of SGA births among women who did not take supplement before pregnancy and those who did not take at all. It is worth mentioning that SGA is one of the main risk factors for adverse outcomes and mortality at birth [72,73], as well as for chronic diseases in later life [74,75,76,77,78]. In line, the implementation of policies based on folic acid supplementation should be one of the main goals to tackle the burden of low birthweight, especially in developing countries. However, there are still controversies about the effect of folic acid supplementation on low birth weight and SGA risks. The majority of studies demonstrated that supplement use before and during pregnancy reduced the risk of SGA [31,36,38,40,42,44,79]. However, others demonstrated an opposite [39,41] or null effect [30,45]. Thus, further research should be encouraged to understand the effect of folic acid supplement use on the risk of SGA and associated outcomes.

Our findings should be interpreted with cautions due to some limitations. Firstly, the Mamma & Bambino study is an ongoing mother-child cohort that recruits pregnant women during their prenatal genetic counselling. For this reason, there is a discrepancy in the number of pregnant women with those who completed pregnancy. We described prevalence and determinants of dietary folate intake and supplement use among 397 pregnant women. Instead, findings on the neonatal effects of folate deficiency were obtained in a subsample of women who completed pregnancy, and thus should be confirmed by future analysis. In general, low sample size in some subgroups (e.g., women with inadequate folate intake and those who completed pregnancy) did not allow us to adjust for potential confounders. Moreover, we cannot rule out the possibility of bias from residual unknown or unmeasured factors. For instance, it has been demonstrated that several genetic polymorphisms involved in folate metabolism affected folate status in healthy subjects before and after folic acid supplement use [80,81]. Secondly, data on dietary folate intake and folic acid supplement use relied on self-reported interviews, which cannot completely exclude reporting errors. For instance, we previously reported that folate intake was higher when assessed with the FFQ than with a 4 days weighed dietary record [13]. To overcome this limitation, in the future, studies should evaluate folate status by measuring folate blood concentration and reviewing the size and morphology of blood cells.

In spite of these limitations, our study underlined the need for improving the adherence of pregnant women with recommendations for dietary folate intake and supplement use. On one hand, this could be achieved with the promotion of a healthy diet rich in vegetables and fruits. On the other hand, the identification of social determinants that might affect the use of folic acid supplements could help the development of public health strategies and policies to reduce the burden of adverse pregnancy outcomes. Although we demonstrated a protective effect of folic acid supplement use on risk of SGA, further research is encouraged to corroborate our findings.

## Figures and Tables

**Table 1:** Characteristics of pregnant women according to folate intake.

Characteristics ^a^	Inadequate Folate Intake (*n* = 257)	Adequate Folate Intake (*n* = 140)	*p*-Value ^b^
**Age, years**	37.0 (5.0)	37.5 (4.0)	0.375
**Educational level**			
Low	19.3%	18.7%	0.214
Medium	51.4%	43.6%	
High	29.3%	37.7%	
**Employed**	56.4%	58.6%	0.679
**Living in couple**	92.3%	93.8%	0.973
**Having children**	68.5%	68.6%	0.985
**Food intolerance (% yes)**	16.7%	10.0%	0.150
**Dietary restriction (% yes)**	19.8%	8.6%	**0.003**
**Smoking status**			
Non-smoker	55.0%	57.6%	**0.028**
Former smoker	15.7%	23.5%	
Smoker	29.3%	18.8%	
**Pregestational BMI, Kg/m^2^**	23.2 (4.9)	22.1 (5.4)	**0.029**
**Pregestational BMI categories**			
Underweight	5.5%	8.6%	0.353
Normal weight	63.7%	66.4%	
Overweight	20.3%	13.6%	
Obese	10.2%	11.4%	
**MDS**	4 (2)	5 (2)	**<0.001**
**Adherence to MD**			
Low	45.1%	20.0%	**<0.001**
Medium	52.9%	63.6%	
High	1.9%	16.4%	
**Preterm birth ^c^**	8.4%	7.2%	0.722
**Birthweight for gestational age**			
SGA	13.7%	4.8%	**<0.001**
AGA	67.4%%	88.4%	
LGA	18.9%	6.9%	

^a^ Results are reported as median (Interquartile range), or percentage. Statistical analysis was performed using Chi-square test for bivariate or categorical variable, and Mann-Whitney test for continuous variables. ^b^ Significant results are indicated in bold font. ^c^ Data are reported for 282 women who completed pregnancy. Abbreviations: BMI, Body Mass Index; MDS, Mediterranean Diet Score.

**Table 2:** Characteristics of folate deficient women according to supplement use.

Characteristics ^a^	Non-Users (*n* = 9)	Insufficient Users (*n* = 188)	Recommended Users (*n* = 60)	*p*-Value ^b^
**Age, years**	38.0 (8.0)	37.0 (4.0)	37.0 (4.0))	0.884
**Educational level**				
Low	44.4%	22.9%	1.7%	**<0.001**
Medium	55.6%	44.1%	40.0%	
High	0%	33.0%	58.3%	
**Employed**	55.6%	52.7%	68.3%	0.103
**Living in couple**	90.1%	91.2%	94.2	0.878
**Having children**	66.7%	70.2%	63.3%	0.603
**Food intolerance (% yes)**	0%	17.0%	18.3%	0.264
**Dietary restriction (% yes)**	33.3%	18.6%	21.7%	0.514
**Smoking status**				
Non-smoker	55.6%	55.1%	66.1%	0.078
Former smoker	33.3%	21.9%	27.1%	
Smoker	11.1%	23.0%	6.8%	
**Pregestational BMI, Kg/m^2^**	22.7 (3.2)	23.6 (5.3)	22.2 (3.9)	0.256
**Pregestational BMI categories**				
Underweight	0%	5.9%	5.0%	0.575
Normal weight	66.7%	60.4%	73.3%	
Overweight	33.3%	20.9%	16.7%	
Obese	0%	12.3%	5.0%	
**MDS**	3 (3)	4 (2)	4 (2)	**0.047**
**Adherence to MD**				
Low	66.7%	46.8%	36.7%	0.424
Medium	33.3%	51.1%	61.7%	
High	0%	2.1%	1.7%	

^a^ Results are reported as median (Interquartile range), or percentage. Statistical analysis was performed using Chi-square test for bivariate or categorical variable, and Kruskal-Wallis test for continuous variables. ^b^ Significant results are indicated in bold font. Abbreviations: BMI, Body Mass Index; MDS, Mediterranean Diet Score.
